# Aluminum-doped ceria-zirconia solid solutions with enhanced thermal stability and high oxygen storage capacity

**DOI:** 10.1186/1556-276X-7-542

**Published:** 2012-10-01

**Authors:** Qiang Dong, Shu Yin, Chongshen Guo, Tsugio Sato

**Affiliations:** 1 Center for Exploration of New Inorganic Materials (CENIM), Institute of Multidisciplinary Research for Advanced Materials, Tohoku University, 2-1-1 Katahira, Aoba-ku, Sendai, 980-8577, Japan

**Keywords:** Solvothermal, Aluminum, Solid solutions, Catalysis, Oxygen storage capacity, Thermal stability

## Abstract

A facile solvothermal method to synthesize aluminum-doped ceria-zirconia (Ce_0.5_Zr_0.5-*x*_Al_*x*_O_2-*x*/2_, *x* = 0.1 to 0.4) solid solutions was carried out using Ce(NH_4_)_2_(NO_3_)_6_, Zr(NO_3_)_3_·2H_2_O Al(NO_3_)_3_·9H_2_O, and NH_4_OH as the starting materials at 200°C for 24 h. The obtained solid solutions from the solvothermal reaction were calcined at 1,000°C for 20 h in air atmosphere to evaluate the thermal stability. The synthesized Ce_0.5_Zr_0.3_Al_0.2_O_1.9_ particle was characterized for the oxygen storage capacity (OSC) in automotive catalysis. For the characterization, X-ray diffraction, transmission electron microscopy, and the Brunauer-Emmet-Teller (BET) technique were employed. The OSC values of all samples were measured at 600°C using thermogravimetric-differential thermal analysis. Ce_0.5_Zr_0.3_Al_0.2_O_1.9_ solid solutions calcined at 1,000°C for 20 h with a BET surface area of 18 m^2^ g^−1^ exhibited a considerably high OSC of 427 μmol-O g^−1^ and good OSC performance stability. The same synthesis route was employed for the preparation of the CeO_2_ and Ce_0.5_Zr_0.5_O_2_. The incorporation of aluminum ion in the lattice of ceria-based catalyst greatly enhanced the thermal stability and OSC.

## Background

Ceria (CeO_2_)-based materials have attracted considerable interest for more than half a century due to their far-ranging applications in catalysts, fuel cells, cosmetics, gas sensors, and solid-state electrolytes and especially their crucial application as promoters of three-way catalysts (TWCs), which are commonly used to reduce the emissions of CO, NO_*x*_, and hydrocarbons from automobile exhausts, because of their excellent oxygen storage capacity (OSC) [1-8]. Since 1990s, CeO_2_-ZrO_2_ solid solutions have gradually replaced pure CeO_2_ as OSC materials in the TWCs to reduce the emission of toxic pollutants (CO, NO_*x*_, hydrocarbons, etc.) from automobile exhaust and because of their enhanced OSC performance and improved thermal stability at elevated temperatures [9-13].

The redox property of CeO_2_ can be greatly enhanced by the incorporation of zirconium ions (Zr^4+^) into the lattice to form a solid solution [14-16]. Nagai et al. have suggested that enhancing the homogeneity of Ce and Zr atoms in the CeO_2_-ZrO_2_ solid solution can improve the OSC performance [17]. The detailed structure and property of CeO_2_-ZrO_2_ solid solutions were reported in a review article by Monte and Kaspar [12]. This review included the results of reducing performance for a series of samples with gradually elevated Ce contents, and a possible mechanism of structural changes in the reducing process was proposed. Fornasiero et al. have reported that an optimum composition like Ce_0.5_Zr_0.5_O_2_ (molar ratio of Ce:Zr = 1:1) can exist as a cubic phase, which can have a considerably high redox property [18]. Using density functional theory, Wang et al. found that in a series of Ce_1-*x*_Zr_*x*_O_2_ solutions with a content of 50%, ZrO_2_ possesses the lowest formation energy of the O vacancy; therefore, Ce_0.5_Zr_0.5_O_2_ exhibits the best OSC performance [19]. Recently, many researchers have paid much attention to prepare the Ce_0.5_Zr_0.5_O_2_ solutions with the homogeneity of the composition, good dispersion of particles, narrow particle size distribution, better crystallinity, and high surface area in order to improve OSC and redox property due to their catalytic applications [20-25].

Although Ce_0.5_Zr_0.5_O_2_ solid solutions have been studied extensively, there are few reports on the preparation of Ce_0.5_Zr_0.5-*x*_M_*x*_O_2-*x*/2_ in the literature [26,27]. Considering the smaller cation radius of Al^3+^ (0.059 nm) compared to those of Zr^4+^ (0.084 nm) and Ce^4+^ (0.097 nm), the incorporation of Al^3+^ into Ce-Zr solid solutions may enhance the oxygen release reaction to form larger Ce^3+^. In the present work, for the first time, we describe the preparation and characterization of Ce_0.5_Zr_0.3_Al_0.2_O_1.9_ solid solutions with high surface area via a facile solvothermal route. The further experiment results show that the introduction of aluminum ion enhances the thermal stability and OSC even after calcination at a very strict condition of 1,000°C for 20 h. The OSC of CeO_2_, Ce_0.5_Zr_0.5_O_2_, and the composites which consisted of different aluminum amounts were also prepared via the same method and compared.

## Methods

All chemicals used were of analytical grade and were purchased from Kanto Chemical Co. Inc., Tokyo, Japan (purity 99.999%). The chemicals were used without further purification.

### Catalysts preparation

The stoichiometric amounts of (NH_4_)_2_Ce(NO_3_)_6_ (6 mmol), ZrO(NO_3_)_2_ (3.6 mmol), and Al(NO_3_)_3_·9H_2_O (2.4 mmol) were dissolved in 60 ml of distilled water. NH_4_OH solution was slowly dropped into the above mixed solution, and the pH value was maintained at 9. The yellow mixed solution was introduced in a 100-ml Teflon®-lined autoclave (SAN-AI Science, Co. Ltd, Nagoya, Japan), which was maintained at 200°C for 24 h, then cooled to room temperature naturally. The obtained products were washed with distilled water three times and dried in air at 100°C for 12 h to form the as-prepared fresh samples. Finally, the fresh samples were calcined at 1,000°C for 20 h in air atmosphere to evaluate the thermal stability. The same synthesis route was employed for the preparation of the CeO_2_ and Ce_0.5_Zr_0.5_O_2_.

### OSC analysis

The OSC of the samples calcined at 1,000°C for 20 h was determined by thermogravimetric-differential thermal analysis (TG-DTA; Rigaku TAS-200, Rigaku Corporation, Tokyo, Japan) at 600°C. Before the measurements, the samples were held in flowing air at 600°C for 30 min to remove residual water and other volatile gases. The mixed gas of CO-N_2_ (100 cm^3^ min^−1^) and air (100 cm^3^ min^−1^) was flowed alternately at 600°C. Finally, OSC was analyzed after getting the TGA profile.

### Characterization

The phase composition of the sample was determined by X-ray diffraction analysis (XRD; Bruker D2 Phaser, Bruker Optik GmbH, Ettlingen, Germany) using graphite-monochromized CuKα radiation. The morphology and size of the samples were determined by transmission electron microscopy (TEM; JEOL JEM-2010, JEOL Ltd., Akishima, Tokyo, Japan). The specific surface area was measured using a BET (NOVA 4200e, Quantachrome GmbH and Co. KG, Odelzhausen, Germany) surface area and pore size analyzer.

## Results and discussion

**Figure 1:**
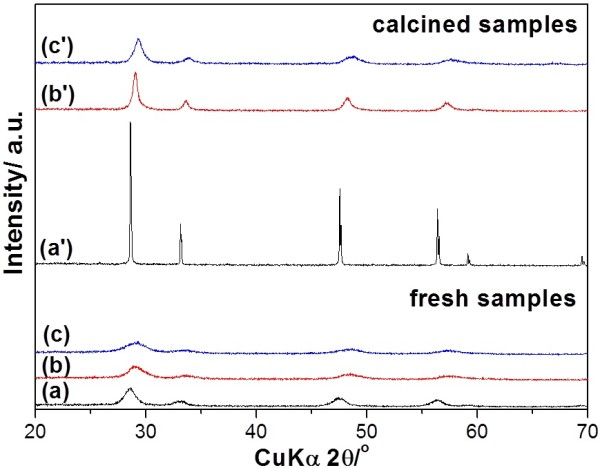
**XRD patterns of fresh and calcined samples. **Fresh samples: (a) CeO_2_, (b) Ce_0.5_Zr_0.5_O_2_, and (c) Ce_0.5_Zr_0.3_Al_0.2_O_1.9_. Calcined samples: (a') CeO_2_, (b') Ce_0.5_Zr_0.5_O_2_, and (c') Ce_0.5_Zr_0.3_Al_0.2_O_1.9_.

All products of (a) CeO_2_, (b) Ce_0.5_Zr_0.5_O_2_, and (c) Ce_0.5_Zr_0.3_Al_0.2_O_1.9_ consisted of a single phase of fluorite structure (Figure 1 (a) to (c)). All the diffraction patterns exhibited broad peaks, suggesting that the fresh samples were nanocrystalline materials. The calcined samples had a slight shift in diffraction peaks when compared to the pure CeO_2_ XRD pattern, indicating the formation of corresponding solid solutions. The calculated lattice parameters of the calcined samples of Ce_0.5_Zr_0.5_O_2_ (*a* = 0.5384 nm) and Ce_0.5_Zr_0.3_Al_0.2_O_1.9_ (*a* = 0.5299 nm) are smaller than that of CeO_2_ (*a* = 0.5413 nm). The shrinkage of lattice cells may be due to the substitution of the smaller cation radius of Zr^4+^ (0.084 nm) and Al^3+^ (0.0059 nm) with Ce^4+^ (0.097 nm). No phase separation was noticed even at such high calcination temperatures at 1,000°C for 20 h, except the increase of particle size (Figure 1 (a') to (c')). The crystal sizes of the fresh CeO_2_, Ce_0.5_Zr_0.5_O_2_, and Ce_0.5_Zr_0.3_Al_0.2_O_1.9_ calculated by Scherer's formula were 9, 5, and 3 nm, while those of the calcined CeO_2_, Ce_0.5_Zr_0.5_O_2_, and Ce_0.5_Zr_0.3_Al_0.2_O_1.9_ were 35, 10, and 8 nm, respectively.

**Figure 2:**
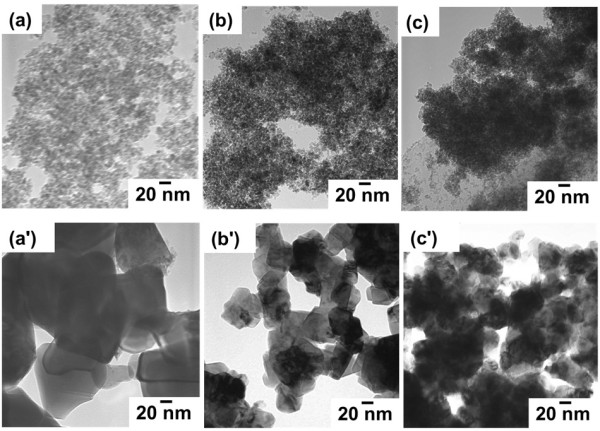
**TEM images of fresh and calcined samples. **Fresh samples: (a) CeO_2_, (b) Ce_0.5_Zr_0.5_O_2_, and (c) Ce_0.5_Zr_0.3_Al_0.2_O_1.9_. Calcined samples: (a') CeO_2_, (b') Ce_0.5_Zr_0.5_O_2_, and (c') Ce_0.5_Zr_0.3_Al_0.2_O_1.9_.

The morphology and size of the fresh and calcined samples (1,000°C for 20 h) were observed by TEM as shown in Figure 2. For the fresh samples, the particles seem to be partly dispersed and formed small agglomerates (Figure 2 (a) to (c)), and the single particle exhibited a spherical-like morphology with the diameters of 9 to 12 nm, 5 to 8 nm, and 3 to 5 nm for CeO_2_, Ce_0.5_Zr_0.5_O_2_, and Ce_0.5_Zr_0.3_Al_0.2_O_1.9_, respectively, which are in agreement with the crystallite size calculated from Scherer's formula. The particle size increased after calcination at 1,000°C for 20 h because of aggregation, and the particle sizes were found to increase to 90 to 100 nm, 50 to 55 nm, and 30 to 35 nm for the CeO_2_, Ce_0.5_Zr_0.5_O_2_, and Ce_0.5_Zr_0.3_Al_0.2_O_1.9_ samples as shown in Figure 2 (a') to (c'), respectively.

**Figure 3:**
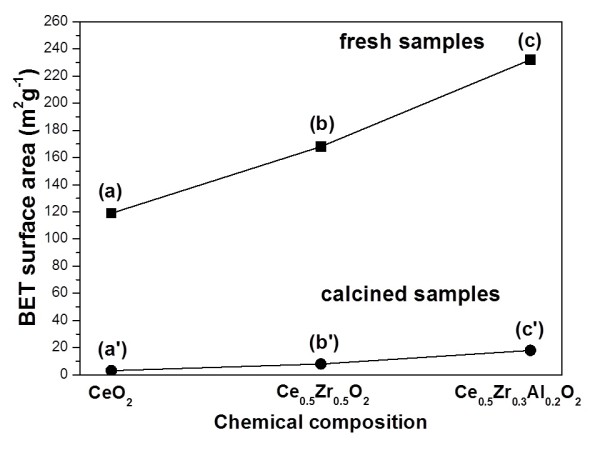
**BET specific surface areas of fresh and calcined samples. ** Fresh samples: (a) CeO_2_, (b) Ce_0.5_Zr_0.5_O_2_, and (c) Ce_0.5_Zr_0.3_Al_0.2_O_1.9_. Calcined samples: (a') CeO_2_, (b') Ce_0.5_Zr_0.5_O_2_, and (c') Ce_0.5_Zr_0.3_Al_0.2_O_1.9_.

BET nitrogen adsorption-desorption analysis was undertaken to measure the specific surface area of all samples. As a result, the fresh sample of Ce_0.5_Zr_0.3_Al_0.2_O_1.9_ showed a much higher surface area (232 m^2^ g^−1^) than those of CeO_2_ (119 m^2^ g^−1^) and Ce_0.5_Zr_0.5_O_2_ (168 m^2^ g^−1^, Figure 3 (a) to (c)). After calcinations at 1,000°C for 20 h in air, the specific surface areas of CeO_2_ (3 m^2^ g^−1^) and Ce_0.5_Zr_0.5_O_2_ (8 m^2^ g^−1^) decreased to less than 10 m^2^ g^−1^, but the sample of Ce_0.5_Zr_0.3_Al_0.2_O_1.9_ exhibited a relatively higher BET specific surface area of 18 m^2^ g^−1^ (Figure 3 (a') to (c')).

**Figure 4:**
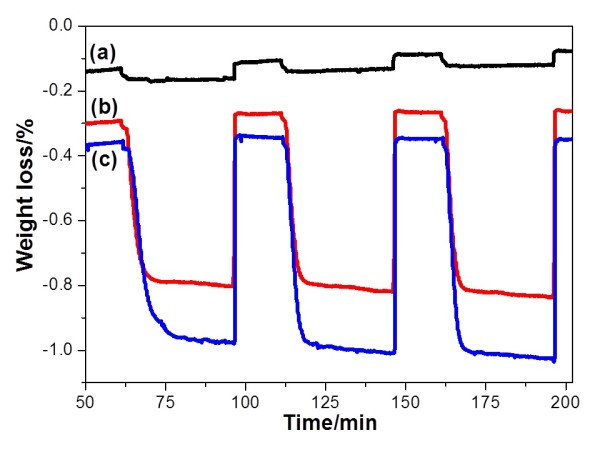
**TG profiles of calcined samples **( **1 **, **000 **° **C **, **20 h **) **at 600 **°**C**, **which show oxygen release **/ **storage properties. **(a) CeO_2_, (b) Ce_0.5_Zr_0.5_O_2_, and (c) Ce_0.5_Zr_0.3_Al_0.2_O_1.9_.

**Figure 5:**
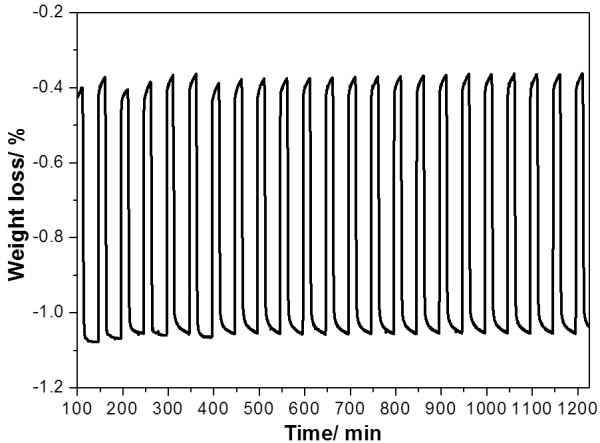
**TG profiles during measurement ofOSC at 600**°**C for Ce**_**0**.**5**_**Zr**_**0**.**3**_**Al**_**0**.**2**_**O**_**1**.**9**_ (**1**,**000**°**C**, **20 h**) **after 22 cycles.** The profiles show oxygen release/storage properties.

The OSC values of the calcined samples were determined at 600°C with a continuous flow of CO-N_2_ gas and air alternately. Figure 4 shows the typical TG profiles of the CeO_2_, Ce_0.5_Zr_0.5_O_2_, and Ce_0.5_Zr_0.3_Al_0.2_O_1.9_ samples. The TG profile shows the oxygen release/storage performance of the CeO_2_, Ce_0.5_Zr_0.5_O_2_, and Ce_0.5_Zr_0.3_Al_0.2_O_1.9_ samples at 600°C with time. As a result, Ce_0.5_Zr_0.3_Al_0.2_O_1.9_ exhibited a higher OSC of 427 μmol-O g^−1^, when compared to those of the CeO_2_ (25 μmol-O g^−1^) and Ce_0.5_Zr_0.5_O_2_ (350 μmol-O g^−1^) samples (Table 1). It is accepted that the OSC is dependent on the specific surface area; it is obvious that Ce_0.5_Zr_0.3_Al_0.2_O_1.9_ exhibited the highest specific surface area and highest OSC values even after calcination at such high temperature as 1,000°C for 20 h. In order to examine OSC performance stability, oxygen release/storage cycle measurement was tested, and Ce_0.5_Zr_0.3_Al_0.2_O_1.9_ retained the same OSC even after 22 cycles (Figure 5). The result indicates that Ce_0.5_Zr_0.3_Al_0.2_O_1.9_ has good OSC performance stability.

**Table 1:** **OSC at 600 **° **C of the CeO **_**2 **_, **Ce **_**0 **.**5 **_**Zr **_**0 **.**5 **_**O**_**2 **_, **and Ce **_**0 **.**5 **_**Zr **_**0 **.**3 **_**Al **_**0 **.**2 **_**O **_**1 **.**9 **_**calcined at 1 **,**000 **° **C for 20 h**

**Chemical composition**	**OSC (μmol-O g **^− **1 **^**)**
CeO_2_	25.0
Ce_0.5_Zr_0.5_O_2_^a^	350.0
Ce_0.5_Zr_0.3_Al_0.2_O_1.9_	427.0

^a^It is accepted that the Ce_0.5_Zr_0.5_O_2_ composition possessed excellent OSC property [10-14].

**Figure 6:**
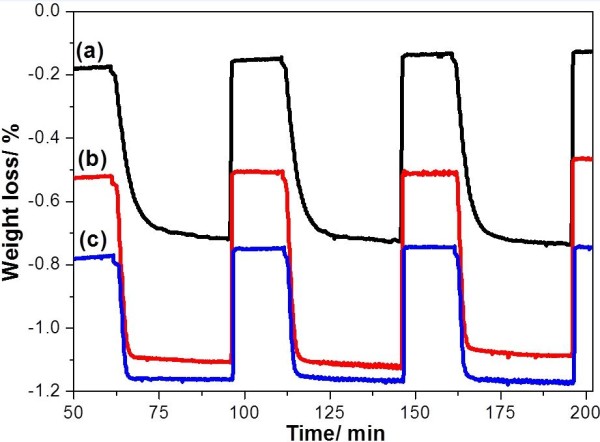
**TG profiles during measurement ofOSC at 600**°**C for calcined samples(1**,**000**°**C**, **20 h).** The profiles show oxygen release/storage properties. (a) Ce_0.5_Zr_0.4_Al_0.1_O_1.95_, (b) Ce_0.5_Zr_0.2_Al_0.3_O_1.85_, and (c) Ce_0.5_Zr_0.1_Al_0.4_O_1.8_.

The amount of incorporated aluminum was also controlled to test its effect on the OSC of the calcined sample as shown in Figure 6 and Table 2. As a result, Ce_0.5_Zr_0.3_Al_0.2_O_1.9_ exhibited the highest OSC of 427 μmol-O g^−1^ (Table 1), when compared to those of the Ce_0.5_Zr_0.4_Al_0.1_O_1.95_ (378 μmol-O g^−1^), Ce_0.5_Zr_0.2_Al_0.3_O_1.85_ (389 μmol-O g^−1^), and Ce_0.5_Zr_0.1_Al_0.4_O_1.8_ (261 μmol-O g^−1^) samples (Table 2), Therefore, in Ce_0.5_Zr_0.5-*x*_Al_*x*_O_*y*_ (0.1 <*x* < 0.5, *x* is the amount of incorporated aluminum), the most appropriate amount of incorporated aluminum might be around *x* = 0.2.

**Table 2:** **OSC at 600 **° **C of the Ce **_**0 **.**5 **_**Zr **_**0 **.**4 **_**Al **_**0 **.**1 **_**O **_**1 **.**95 **_, **Ce **_**0 **.**5 **_**Zr **_**0 **.**2 **_**Al **_**0 **.**3 **_**O**_**1 **.**85 **_, **and Ce **_**0 **.**5 **_**Zr **_**0 **.**1 **_**Al **_**0 **.**4 **_**O **_**1 **.**8 **_**calcined at 1 **, **000 **° **C for 20 h**

**Chemical composition**	**OSC (μmol-O g **^− **1 **^**)**
Ce_0.5_Zr_0.4_Al_0.1_O_1.95_	378.0
Ce_0.5_Zr_0.2_Al_0.3_O_1.85_	389.0
Ce_0.5_Zr_0.1_Al_0.4_O_1.8_	261.0

## Conclusions

Ce_0.5_Zr_0.3_Al_0.2_O_1.9_ solid solutions with high surface area were successfully synthesized via a facile solvothermal method. The structures of the fresh samples and calcined samples were characterized by X-ray diffraction. The lattice parameters of the Ce_0.5_Zr_0.3_Al_0.2_O_1.9_ solid solution are smaller than those of CeO_2_ and Ce_0.5_Zr_0.5_O_2_, suggesting the incorporation of the Al^3+^ into Ce-Zr solid solutions. The fresh particles showed spherical-like morphology with a diameter of 3 to 5 nm determined by TEM. The Ce_0.5_Zr_0.3_Al_0.2_O_1.9_ solid solutions exhibited a remarkably higher oxygen storage capacity than those of the CeO_2_ and Ce_0.5_Zr_0.5_O_2_ samples prepared via the same method, even after calcination at 1,000°C for 20 h, indicating the improvement of the OSC and thermal stability due to the incorporation of aluminum. An appropriate amount of incorporated aluminum is also suggested.

## Competing interests

The authors declare that they have no competing interests.

## Authors’ contributions

QD participated in the design of the study, carried out the total experiments, and performed the result analysis as well as drafted the manuscript. SY participated in the design of the study, gave the theoretical and experimental guidance, and made the corrections of manuscript. CG mainly helped in the experiments and measurements. TS gave the theoretical and experimental guidance and helped to amend the manuscript. All authors read and approved the final manuscript.

## Authors’ information

QD, SY, CG, and TS are an assistant professor, an associate professor, a Ph.D. candidate, and a full professor, respectively, at the Institute of Multidisciplinary Research for Advanced Materials, Tohoku University.
